# Perioperative Blood Transfusion Impairs Overall Survival Following Radical Resection for Colorectal Cancer: A Propensity Score-Matched Analysis

**DOI:** 10.3390/cancers18081198

**Published:** 2026-04-09

**Authors:** Xiaoran Wang, Zesong Meng, Guangjun Wang, Guiying Wang, Lihua Liu

**Affiliations:** 1 The Second Department of General Surgery, The Fourth Hospital of Hebei Medical University, Shijiazhuang 050011, China; 48701660@hebmu.edu.cn (X.W.); mengzesongming@163.com (Z.M.); hbydwgj@163.com (G.W.); 2 Department of General Surgery, The Second Hospital of Hebei Medical University, Shijiazhuang 050000, China; 3 Department of Tumor Immunotherapy, The Fourth Hospital of Hebei Medical University, Shijiazhuang 050035, China

**Keywords:** colorectal cancer, perioperative blood transfusion, propensity score matching, overall survival, prognostic factor, dose–response relationship

## Abstract

Background/Objectives: Perioperative blood transfusion (BTF) remains controversial regarding its impact on oncological outcomes in colorectal cancer (CRC). This study aimed to evaluate the association between BTF, transfusion volume, and long-term prognosis in CRC patients undergoing radical resection. Methods: We conducted a retrospective cohort study of 1777 CRC patients who underwent radical surgery at the Fourth Hospital of Hebei Medical University between December 2007 and April 2015. Propensity score matching (PSM) was applied to minimize selection bias between BTF and non-BTF groups. Logistic regression identified factors associated with BTF receipt. Cox proportional hazards models assessed the association between BTF and 5-year overall survival (OS). X-tile analysis determined optimal cut-off values for transfusion volume stratification. Results: Among 1777 patients, 729 (41.02%) received BTF. After PSM, 524 well-matched pairs showed balanced baseline characteristics. Intestinal obstruction was independently associated with BTF requirement (*p* < 0.001). The BTF group demonstrated significantly inferior 1-, 3-, and 5-year OS compared with non-BTF groups in both overall and PSM cohorts (all *p* < 0.01). Multivariate Cox analysis identified BTF as an independent adverse prognostic factor (HR = 1.44, 95% CI 1.09–1.89, *p* = 0.01). X-tile analysis stratified patients into non-BTF, small-volume (≤4 units), and massive-volume (>4 units) groups. Massive-volume transfusion showed the poorest survival outcomes (*p* < 0.0001) and was independently associated with worse OS (HR = 1.61, 95% CI 1.18–2.20, *p* = 0.003). Supplementary analyses indicated that no independent association was observed between survival outcomes and either preoperative inflammatory markers or the specific timing and type of transfusion. Conclusions: BTF, particularly when exceeding 4 units, independently predicts inferior long-term survival in CRC patients following radical resection. These findings support the implementation of restrictive transfusion strategies in perioperative CRC management. Given its retrospective observational design, this study shows an association but does not establish causality, and our findings should be interpreted in light of the aforementioned limitations.

## Simple Summary

Perioperative blood transfusion, the transfer of blood or blood products during or around the time of surgery, is common in colorectal cancer operations. However, its impact on long-term survival remains unclear. This study investigated whether receiving blood transfusions during surgery affects survival outcomes in colorectal cancer patients. We analyzed data from 1777 patients who underwent radical surgery, comparing those who received transfusions with those who did not. Our findings showed that patients receiving blood transfusions, especially more than four units, had significantly worse long-term survival compared to non-transfused patients. These results suggest that doctors should carefully consider whether to give blood transfusions during perioperation of colorectal cancer and use restrictive strategies when possible to improve patient outcomes.

## 1. Introduction

According to the latest data from the National Cancer Center of the United States, colorectal cancer (CRC) ranks third in both estimated new cases and deaths [1]. In contrast, data from China indicate that CRC is the second most common cancer in terms of new cases and the leading cause of cancer-related mortality [2]. By 2022, China was estimated to report approximately 592,232 new CRC cases and 309,114 CRC-related deaths [3]. While the incidence of CRC is on the rise in China, it has recently declined in the United States [4]. Notably, the CRC incidence rate in China is higher than the average observed in other Asian countries [5]. Primary treatment modalities for CRC include surgery, adjuvant chemotherapy, radiotherapy, and other integrated approaches, among which radical resection (R0 resection) plays a pivotal role in reducing mortality risk [6].

Perioperative blood transfusion (BTF) is frequently required to address preoperative anemia or manage perioperative bleeding [7], and its timely administration is crucial to avoid hemodynamic instability and ensure adequate oxygen delivery during the perioperative period. However, accumulating evidence suggests that perioperative BTF may be associated with increased postoperative complications, prolonged hospitalization, and adverse impacts on prognosis [8,9]. Specifically, BTF has been linked to a higher risk of infection and potentially worse tumor outcomes [10,11], which may be attributed to tumor cell dissemination, surgery-induced inflammation, and the immunosuppressive effects of allogeneic blood transfusion [12,13]—a phenomenon known as transfusion-related immune modulation (TRIM) [14]. Nevertheless, other studies have failed to demonstrate a significant effect of perioperative BTF on CRC prognosis [7,15,16]. These conflicting findings may stem from variations in study design, patient demographics, transfusion thresholds, and unadjusted confounding variables.

To address these inconsistencies and minimize selection bias, we conducted a retrospective cohort study involving CRC patients who underwent radical resection at the Fourth Hospital of Hebei Medical University. Utilizing propensity score matching (PSM), we adjusted for known confounders between patients who did and did not receive perioperative BTF, thereby aiming to provide a more precise evaluation of the independent impact of BTF and transfusion volume on long-term oncological outcomes.

## 2. Materials and Methods

### 2.1. Design and Patients

**Figure 1:**
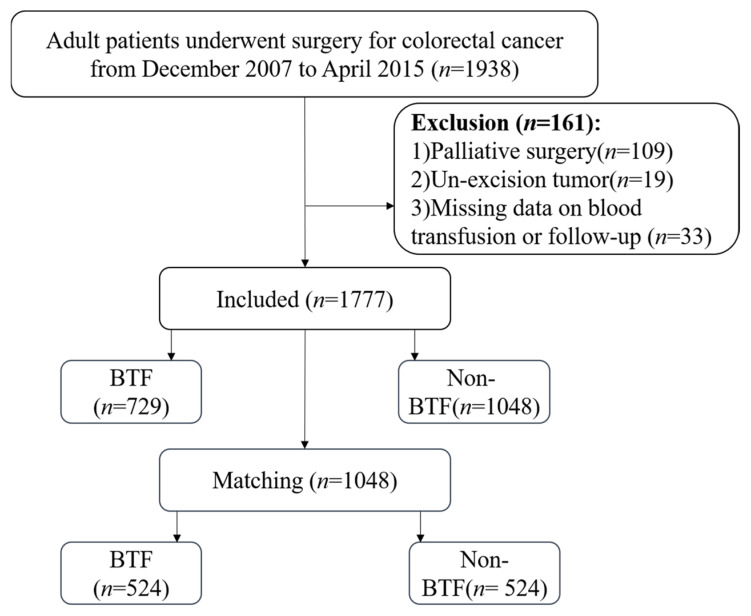
Study flowchart of patient enrollment and propensity score matching.

A total of 1938 consecutive adult patients (≥18 years old) who underwent surgical resection for histologically confirmed colorectal cancer between December 2007 and April 2015 at the Fourth Hospital of Hebei Medical University were screened for eligibility. Exclusion criteria: (1) Patients underwent palliative surgery, (2) Patients with un-excision tumor, (3) Patients with missing data on blood transfusion or follow-up. The exclusion criteria and study flowchart are delineated in Figure 1. Ultimately, data from 1777 patients were included in this retrospective cohort analysis. Patients were stratified based on their receipt of BTF. Ethical approval was obtained from the Medical Ethics Committee of the Fourth Hospital of Hebei Medical University, and written informed consent was acquired from all participants.

### 2.2. Variables and Outcomes

Data were sourced from the electronic clinical information system of the Fourth Hospital of Hebei Medical University, encompassing baseline characteristics and perioperative outcomes of patients undergoing CRC surgery. Baseline variables included demographic factors (sex, age), comorbidities (diabetes, hypertension, coronary atherosclerotic heart disease, old cerebral infarction), tumor-related parameters (tumor size, T stage, N stage, M stage, pTNM stage, tumor site, tumor location, pathological type, tumor thrombus), surgical factors (combined multiorgan resection, surgical type), and other clinical indicators (hospitalization days, family history, co-occurrence of other tumors, obstruction, and mortality).

This study analyzed perioperative transfusion records of packed red blood cells (with or without leukocyte depletion), plasma, or platelets. To further explore the impact of transfusion timing, the administration of all blood products was categorized by timing into three periods: preoperative (from admission to the initiation of surgery), intraoperative (from the initiation to the completion of surgery), and postoperative (from the completion of surgery to hospital discharge).

To evaluate potential residual confounding, we retrospectively collected supplementary data on patients’ perioperative inflammatory markers (e.g., white blood cell count and neutrophil count) and recorded the incidence of major perioperative complications.

### 2.3. Definition of Perioperative Blood Transfusion (BTF)

Perioperative blood transfusion was defined as the administration of blood products, including packed red blood cells, plasma, or platelets, from hospital admission to discharge. Transfusion criteria of packed red blood cells were generally stipulated a hemoglobin level < 80 g/L; patients with hemoglobin levels between 80 and 100 g/L received transfusions based on age (>65 years), cardiovascular or respiratory comorbidities, oxygen consumption, and blood loss rates. Plasma transfusions were administered in accordance with routine clinical transfusion guidelines for coagulation disorders and active bleeding. Platelet transfusions were administered according to standard clinical guidelines, primarily for thrombocytopenia and bleeding risk. 

### 2.4. Propensity Score Matching (PSM)

To mitigate selection bias, patients were categorized into BTF and non-BTFgroups via propensity score matching (PSM) according to the method described by Rubin et al. Propensity scores were computed using variables exhibiting significant inter-group differences, including gender, tumor size, hospitalization days, T stage, N stage, M stage, pTNM stage, combined multiorgan resection, tumor site, tumor location, surgical type, and co-occurrence of other tumors. Nearest neighbor matching (1:1 ratio) without replacement was performed with a caliper width set at 0.05 standard deviation (SD).

### 2.5. Statistical Analysis

Data were analyzed using SPSS (Version 25; IBM, Armonk, NY, USA) and R software (Version 3.4.4, R Foundation for Statistical Computing, Vienna, Austria). Baseline characteristics were presented as counts and percentages (%) for categorical variables, and mean ± SD for continuous variables. Associations between preoperative anemia and clinicopathological features were assessed via *t*-tests (continuous variables) and Chi-squared tests (categorical variables). Spearman’s correlation was used to analyze the relationship between preoperative and postoperative anemia. Kaplan–Meier curves were constructed to depict OS, with survival differences between the BTF and non-BTF groups (pre- and post-PSM) assessed using the log-rank test. Multivariate Cox proportional hazards regression models were used to adjust for prognostic factors potentially impacting OS, with statistical significance set at *p* < 0.05. Additionally, X–tile software (Version 3.6.1) was used to determine the optimal cutoff value for blood transfusion volume in relation to OS among CRC patients.

### 2.6. Postoperative Follow-Up and Surveillance

All patients were routinely followed up according to a standardized protocol at our institution. The surveillance schedule was as follows: clinical examination, laboratory tests (including carcinoembryonic antigen [CEA]), and cross-sectional imaging (computed tomography [CT] of the chest, abdomen, and pelvis) were performed every 3–6 months for the first 2 years, and every 6–12 months thereafter until 5 years postoperatively. A colonoscopy was conducted within the first year after surgery and repeated every 1–3 years as clinically indicated. Tumor recurrence was diagnosed based on either: (1) radiographic evidence of new lesions consistent with recurrence or metastasis on CT or magnetic resonance imaging, confirmed by multidisciplinary team review; or (2) histopathological confirmation via biopsy. Overall survival (OS) was defined as the time from the date of surgery to the date of death from any cause or the last follow-up.

## 3. Results

### 3.1. Characteristics of Patients and Blood Transfusion

This study initially included 1938 patients diagnosed with CRC, of whom 1777 met the inclusion criteria and were enrolled in the final analysis. Among the enrolled patients, 729 (41.02%) received perioperative blood transfusion (BTF group), and the remaining 1048 (58.98%) formed the non-blood transfusion (non-BTF group) (Figure 1). Patient-, operation-, and tumor-related variables, including gender, tumor size, hospitalization days, T stage, N stage, M stage, pTNM stage, combined multiorgan resection, tumor site, tumor location, surgical type, co-occurrence of other tumors and obstruction, varied significantly between the two groups (all *p* < 0.05); (Table 1). 

### 3.2. Risk of Perioperative Blood Transfusion

Multivariate logistic regression analysis was performed to identify independent risk factors for perioperative BTF in patients undergoing radical resection of colorectal cancer. The results indicated that gender, tumor size, length of hospital stay, combined multiorgan resection, tumor site, co-occurrence of other tumors, and intestinal obstruction were independent risk factors for BTF (all *p* < 0.05). Detailed information regarding the multivariate analysis of potential predictors for BTF in the total cohort is presented in Table 2.

### 3.3. Propensity Score Matching to Minimize Selection Bias

To mitigate selection bias, after 1:1 PSM, a total of 524 patient pairs were included for subsequent analysis. Comprehensive balance assessment confirmed that all critical baseline, procedural, and oncological variables were well balanced between the two study groups, with no statistically significant differences observed (all *p* > 0.05). Comparative analysis revealed no significant discrepancies in demographic characteristics, surgical approaches, or tumor features between the BTF group and the non-BTF group. The clinicopathological characteristics of patients after PSM are summarized in Table 1, except for intestinal obstruction, which remained significantly different between the two groups (odds ratio [OR] = 1.91, 95% confidence interval [CI] 1.32–2.75, *p* < 0.001). Specifically, 52 patients (9.9%) in the non-BTF group presented with preoperative intestinal obstruction, whereas the incidence of preoperative obstruction was significantly higher in the BTF group (91 patients, 17.4%). This between-group difference was statistically significant, as detailed in Table 1. Multivariate logistic regression analysis identified intestinal obstruction as an independent risk factor for BTF in CRC patients (OR = 2.06, 95% CI 1.40–3.02, *p* < 0.001), with detailed results presented in Table 2.

To explore potential residual confounding, we analyzed the associations between available systemic inflammatory markers and the requirement for perioperative blood transfusion. Logistic regression analyses showed that white blood cell count, neutrophil count, and their derived indices (e.g., neutrophil-to-white blood cell ratio) exhibited no statistically significant correlations with transfusion need (all *p* > 0.05). Detailed results are presented in Table S1 of the Supplementary Materials. Nutritional indicators (e.g., serum albumin) were not included in the current analyses due to a high rate of missing and inconsistent retrospective records. In addition, we attempted to investigate the relationships between perioperative complications (such as infection and anastomotic leakage) and transfusion. However, owing to the small number of events for each specific complication, a valid multivariable statistical assessment could not be performed. 

### 3.4. Long-Term Survival Analysis in CRC Patients

**Figure 2:**
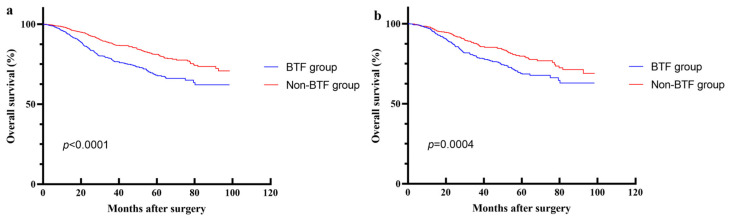
Survival curves of the blood transfusion (BTF) and non-blood transfusion (non-BTF) groups. (**a**) Overall survival in the entire cohort (*p* < 0.0001, log-rank test). (**b**) Overall survival in the propensity score-matched (PSM) cohort (*p* = 0.0004, log-rank test).

During the study follow-up period, the mortality rate in the non-BTF group was 16.9% (169 deaths), whereas the BTF group had a significantly higher mortality rate of 25.2% (184 deaths). Regarding overall survival (OS), the non-BTF group achieved superior 1-, 3-, and 5-year OS rates (97.7%, 87.9%, and 80.8%, respectively) compared with the BTF group (94.8%, 78.4%, and 67.7%, respectively), with a statistically significant difference (*p* < 0.0001) (Figure 2a).

Univariate survival analysis of the entire study cohort demonstrated that BTF was a significant prognostic factor for decreased OS, with a hazard ratio (HR) of 1.816 (95% CI 1.473–2.239, *p* < 0.001), as detailed in Table 3.

### 3.5. Post-Propensity Score Matching Survival Outcomes

**Figure 3:**
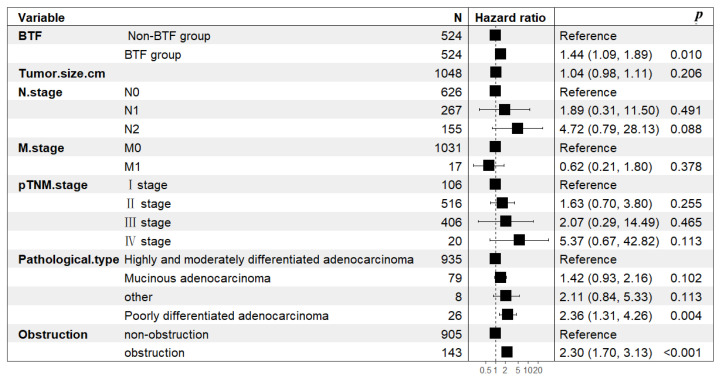
Forest plot of multivariate analysis for prognostic factors of overall survival following radical resection of colorectal cancer in the propensity score-matched cohort.

Following PSM, the 1-, 3-, and 5-year overall survival (OS) rates in the non-BTF group were 97.3%, 87.1%, and 79.3%, respectively. These rates were statistically significantly higher than those in the BTF group (96.2%, 79.6%, and 42.9%, respectively; *p* = 0.0004) (Figure 2b). Univariate survival analysis further identified BTF as a significant prognostic factor influencing OS, with a HR of 1.563 (95% CI 1.193–2.048, *p* = 0.001). Multivariate Cox proportional hazards regression analysis, after adjusting for potential confounding factors, confirmed that BTF was an independent predictor of decreased OS (HR = 1.44, 95% CI 1.09–1.89, *p* = 0.01) (Figure 3).

### 3.6. Linear Regression Analysis of Blood Transfusion Volume and Prognosis

**Figure 4:**
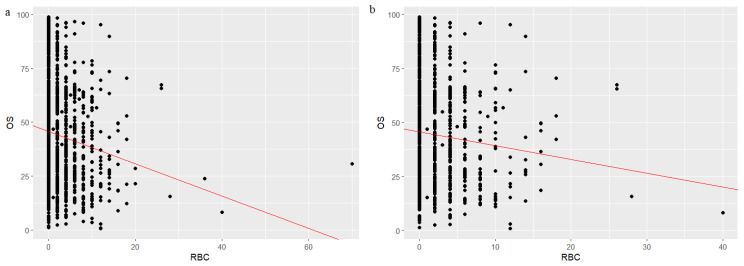
Linear regression analysis of blood transfusion volume and prognosis in colorectal cancer (CRC) patients in the total cohort (**a**) and propensity score-matched cohort (**b**). The red regression line exhibits a downward trend, suggesting that increased red blood cell transfusion volume correlates with decreased overall patient survival.

A linear regression analysis was performed to explore the association between blood transfusion volume and prognosis in CRC patients. The results revealed a significant correlation between blood transfusion volume and patient prognosis, with an increased blood transfusion volume being significantly associated with a poorer prognosis (*p* < 0.05). Specifically, the regression coefficients (β) were −0.749 and −0.6391 in the total cohort and PSM cohort, respectively, indicating that an increase in blood transfusion volume was associated with a corresponding reduction in the probability of a favorable prognosis in both cohorts (all *p* < 0.01) (Figure 4). This finding highlights the clinical significance of minimizing perioperative blood transfusion to potentially improve long-term outcomes in CRC patients.

### 3.7. Perioperative Blood Transfusion Volume Influences Overall Survival in CRC Patients

X-tile software was used to analyze the association between BTF volume and OS in CRC patients. This software iteratively tests various cutoff values to identify the optimal threshold that best stratifies patients into distinct prognostic subgroups. A 4-unit cutoff value was identified as the optimal threshold, which maximized the separation of survival curves and indicated a significant difference in prognosis between patients who received <4 units and those who received ≥4 units of blood transfusion. Based on this cutoff, patients were categorized into three distinct groups according to blood transfusion volume: the non-blood transfusion (non-BTF) group, the small-volume blood transfusion (SBTF) group (≤4 units), and the massive-volume blood transfusion (MBTF) group (>4 units).

**Figure 5:**
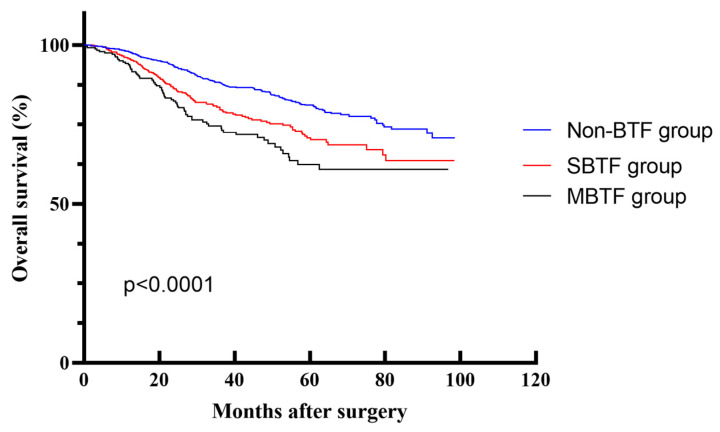
Overall survival curves of patients in the SBTF, MBTF and non-BTF groups.

With respect to OS, the non-BTF group exhibited superior 1-, 3-, and 5-year OS rates of 97.7%, 87.9%, and 80.8%, respectively, which were significantly higher than those in the SBTF group (95.5%, 80.2%, and 70.2%) and the MBTF group (93.3%, 73.9%, and 60.8%) (*p* < 0.0001) (Figure 5). Patients in the MBTF group with a transfusion volume exceeding 4 units displayed the worst OS. X-tile analysis identified the 4-unit transfusion threshold as the critical cutoff that effectively stratified patients into distinct prognostic subgroups. These findings emphasize the importance of blood transfusion volume as a key determinant in clinical management and survival prediction.

**Figure 6:**
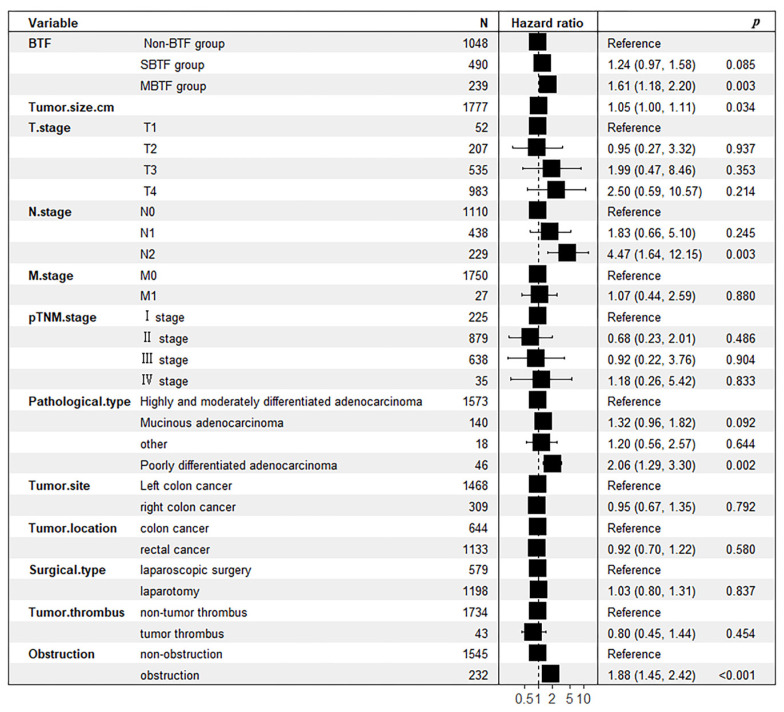
Forest plot of multivariate analyses for prognostic factors of overall survival following radical resection of colorectal cancer.

Univariate analysis across the entire cohort demonstrated that SBTF and MBTF were significant prognostic factors for inferior OS, with HR of 1.642 (95% CI: 1.296–2.080, *p* < 0.001) and 2.222 (95% CI: 1.675–2.947, *p* < 0.001), respectively (Table 4). Following adjustment for confounding factors in multivariate Cox proportional hazards regression, MBTF remained an independent prognostic factor for reduced OS (HR = 1.61, 95% CI: 1.18–2.20, *p* = 0.003) (Figure 6).

To comprehensively evaluate the impact of perioperative blood transfusion, we further analyzed the associations of red blood cell transfusion and plasma transfusion at different administration timings (preoperative, intraoperative, and postoperative) with overall survival. As shown in Tables S2 and S3 in the Supplementary Materials, none of the preoperative, intraoperative, or postoperative red blood cell transfusions were associated with a statistically significant hazard ratio (all *p* > 0.05). Similarly, plasma transfusion at any timing was not significantly associated with overall survival (all *p* > 0.05). Owing to the low incidence, platelet transfusion was not included in separate timing-stratified analyses. These results indicate that, in our study cohort, neither transfusion of specific blood products nor transfusion at specific time points was an independent prognostic factor for long-term patient survival.

## 4. Discussion

Radical surgery remains a cornerstone in the management of CRC [17,18], with total rectal resection for rectal cancer and total colon resection for colon cancer being the standard surgical approaches [19]. Over the past decade, outcomes of radical CRC surgery have been significantly improved owing to the adoption of multiple operative and non-operative strategies (e.g., low central venous pressure [CVP] anesthesia, autologous blood transfusion, antifibrinolytics/anticoagulants). Concurrently, there has been a growing emphasis on restrictive blood transfusion practices during colorectal surgery across all clinical indications [20].

Perioperative BTF is well recognized to affect various postoperative outcomes, including complication rates, hospital length of stay, short-term mortality, and long-term prognosis [7,8,21,22]. The present study explored the factors associated with BTF in CRC surgery, employing PSM to mitigate baseline information bias and control for potential confounding variables.

Post-PSM analysis provided a more precise assessment of the independent risk factors for BTF, including gender, tumor size, hospital length of stay, multiple organ resection, tumor location, coexisting tumors, and preoperative obstruction. Notably, preoperative hemoglobin level and intraoperative blood loss were identified as independent risk factors for BTF following PSM. Anemia, blood loss, and transfusion can be regarded as the “three evils” that adversely affect patient mortality, with intricate interrelationships among them [23,24,25]. A key objective of this study was to explore the interplay between preoperative anemia and intraoperative transfusion. Our findings indicated that both preoperative anemia and intraoperative transfusion were associated with patient OS; however, multivariate Cox regression analysis revealed that preoperative hemoglobin levels were not an independent prognostic factor for OS. Despite this, the combination of preoperative anemia and transfusion was associated with a higher risk of death and cancer progression compared with patients without transfusion. These results suggest that the use of intraoperative blood transfusion for the management of anemia requires careful consideration, and strategies targeting anemia tolerance and appropriate restriction of blood transfusion may contribute to improving the prognosis of CRC patients.

The relationship between perioperative BTF and prognosis following CRC surgery remains controversial [26,27]. A recent systematic review synthesized available evidence regarding the potential links between BTF with postoperative complications and long-term tumor outcomes in radical CRC surgery. Ten included studies specifically investigated the correlation between BTF and colorectal cancer liver metastasis, yet findings across these studies were inconsistent [28]. All included studies were single-institutional with moderate to severe bias. Cannon et al. [29] reported that BTF was independently associated with postoperative non-bleeding complications but did not adversely affect disease-free survival (DFS) or OS. In contrast, Shiba et al. [30] and Sultana et al. [31] identified a negative correlation between BTF and OS in unadjusted analyses. Meanwhile, Schiergens et al. [32] found that BTF was associated with reduced recurrence-free survival (RFS), and the latter study noted a decreased 5-year OS rate after excluding 90-day postoperative mortality. The present study highlights the significance of BTF as an independent prognostic factor for OS in CRC patients, suggesting that transfusion volume may exert a substantial influence on tumor prognosis.

The immunomodulatory effects of BTF offer a potential biological mechanism underlying these observations. Allogeneic blood transfusion may alter immune system receptor responses, thereby affecting tumor growth and recurrence [33,34]. Furthermore, “storage lesions” in transfused blood may compromise post-transfusion outcomes, such as increasing the risk of infection and multiple organ failure. In the recent study, patients were stratified into non-BTF, SBTF, and MBTF groups based on total transfusion volume, and a dose-dependent relationship between BTF volume and OS was observed. This finding supports the hypothesis that the physiological impact of BTF on the host is proportional to the volume of blood transfused. Specifically, the MBTF group exhibited the lowest OS rate, indicating a threshold effect where the risk associated with BTF increases significantly beyond a certain transfusion volume.

While our PSM-adjusted and multivariate analyses identified perioperative blood transfusion (BTF), particularly massive-volume transfusion (>4 units), as an independent predictor of inferior OS, the interpretation of this association requires careful consideration with regard to causality. A fundamental methodological challenge in observational studies of transfusion outcomes is indication bias or confounding by severity. Specifically, the decision to transfuse is not random but represents a clinical necessity for patients with severe anemia, significant intraoperative blood loss, or poorer physiological reserve. Consequently, patients in the BTF group likely represented a cohort with inherently greater tumor burden, more complex surgeries, or worse baseline status—all factors independently associated with worse prognosis. Although we employed PSM to balance a comprehensive set of measurable confounders (including tumor stage, size, surgical procedure, and intraoperative blood loss) and further adjusted for these variables in multivariate Cox models, thereby strengthening the inference that BTF is an independent risk factor (PSM cohort: HR = 1.44, 95% CI 1.09–1.89), we cannot entirely rule out residual confounding from unmeasured or unmeasurable variables. Therefore, the observed association may partly reflect the biological effects of transfusion itself and partly serve as a marker of the underlying critical condition that necessitated transfusion. Nonetheless, the significant dose–response relationship between transfusion volume and survival outcome (with the worst survival in the MBTF group: HR = 1.61, 95% CI 1.18–2.20), which persisted after multivariable adjustment, supports a potential causal contribution of transfusion exposure. Future prospective studies or those employing more sophisticated causal inference methods are warranted to further disentangle this complex relationship.

The underlying biological mechanisms linking BTF to inferior survival remain to be fully elucidated. A widely discussed and plausible hypothesis is transfusion-related immunomodulation (TRIM). Previous studies suggest that allogeneic blood transfusion may alter the recipient’s immune status through various pathways, known as the TRIM effect [12,13]. The significant dose–response relationship between transfusion volume and survival outcome observed in our study is consistent with the hypothesis of a biological gradient for a TRIM effect. Our study observed that blood transfusion, particularly high-volume transfusion, is an independent adverse prognostic factor for survival. Although the underlying biological mechanisms remain incompletely elucidated, they are likely closely related to transfusion-related immunomodulation (TRIM). Allogeneic blood transfusion introduces foreign antigens and bioactive substances, which may affect the host immune system through multiple pathways. First, it suppresses the function of effector immune cells, such as natural killer cells and cytotoxic T lymphocytes, thereby impairing antitumor immune surveillance [12,13]. Second, transfusion may trigger the release of pro-inflammatory cytokines and activate regulatory T cells, creating an immunosuppressive microenvironment that facilitates the survival and proliferation of tumor cells [12,13]. In addition, storage lesion products generated during the preservation of packed red blood cells can further exacerbate systemic inflammation and oxidative stress [12,13]. Collectively, these immunomodulatory and inflammatory alterations constitute a plausible biological framework explaining why blood transfusion is associated with elevated tumor recurrence and reduced long-term survival. However, it is important to note that as a retrospective study, we did not prospectively collect serial blood samples from patients for analysis of direct immunological biomarkers (e.g., immune cell subsets, cytokine profiles) before and after transfusion. Therefore, we cannot provide laboratory evidence confirming the occurrence of TRIM in our cohort. Future prospective studies incorporating systematic biospecimen collection and immunological assays peri-transfusion are warranted to directly test whether TRIM plays a causal role in the clinical association observed here.

A point warranting specific discussion is that despite PSM achieving balance for most baseline covariates, “intestinal obstruction” remained imbalanced between groups in the matched cohort (17.4% in BTF vs. 9.9% in non-BTF). Intestinal obstruction is a serious complication of CRC, associated with more advanced disease, poorer nutritional status, and more complex surgery, and is itself a well-established negative prognostic factor. Consequently, this residual imbalance represents a potential limitation of our study, implying that a portion of the observed survival disparity might be attributable not solely to transfusion but to the worse baseline condition signified by obstruction. This residual confounding could potentially lead to an overestimation of the detrimental effect of BTF. Several points, however, support the robustness of our primary finding. First, PSM successfully balanced the vast majority of known important prognostic variables, including tumor TNM stage, size, and extent of surgery, thereby substantially reducing confounding. Second, we identified a significant dose–response relationship between transfusion volume and survival (with the worst outcomes in the massive-volume > 4 units group). This gradient effect is less readily explained by the dichotomous variable “obstruction” and lends stronger support to a biological effect of the transfusion exposure per se. Finally, in the multivariate Cox analysis, BTF remained an independent risk factor for OS even within the matched cohort. Nonetheless, we acknowledge that the incomplete balancing of obstruction necessitates a cautious interpretation of the conclusion that “BTF is an independent adverse prognostic factor” within this context. Future studies may require more sophisticated matching approaches or larger samples to better control for this strong confounder.

While the hazard ratios associated with blood transfusion (HR = 1.3–1.6) in this study are modest in magnitude, their clinical and public health implications are substantial. First, from a population perspective, given the high incidence of CRC in China [2,3] and the high prevalence of transfusion (41%) in our cohort, even a relative risk increase in HR = 1.3–1.4 would translate into a considerable number of attributable deaths, significantly impacting disease burden. Second, the absolute risk difference is more clinically intuitive: in the matched cohort, the 5-year overall survival rate differed by a striking 36.4 percentage points (79.3% vs. 42.9%) between groups, a difference considered large in oncology. Third, a clear dose–response relationship was observed: the HR increased to 1.61 for massive transfusion (>4 units), indicating that the risk is not fixed or minimal but escalates significantly with volume, providing a clear clinical threshold for intervention (i.e., avoiding > 4 units). Finally, and most critically, perioperative transfusion is a treatment decision fully under clinician control and modifiable. Compared to non-modifiable prognostic factors (e.g., age, tumor stage), optimizing a modifiable factor with a demonstrated dose-dependent risk (transfusion) represents one of the most cost-effective and actionable strategies to improve patient outcomes. Therefore, even a moderately elevated relative risk strongly supports the implementation of a more restrictive transfusion strategy in clinical practice.

While our findings strongly advocate for a restrictive transfusion policy, it is acknowledged that transfusion remains unavoidable for a subset of patients with critical anemia or life-threatening bleeding. For these cases, strategies to mitigate potential transfusion-related risks should be considered. First, the use of leukoreduced blood products has been proposed to reduce the load of donor leukocytes, potentially attenuating transfusion-related immunomodulation (TRIM) and associated adverse effects. Second, adhering strictly to a restrictive transfusion threshold (e.g., hemoglobin < 7–8 g/dL in stable patients), as opposed to a liberal strategy, is itself a key risk-mitigation approach, ensuring that only patients with a clear physiologic need receive transfusion. Third, proactive management of preoperative anemia (e.g., with iron supplementation or erythropoiesis-stimulating agents where appropriate) in the weeks before elective surgery can reduce the likelihood of requiring allogeneic transfusion. Implementing such a patient blood management program represents a comprehensive strategy to minimize exposure to allogeneic blood and its potential downsides.

This study further revealed that neither the timing (preoperative, intraoperative, postoperative) of red blood cell transfusion nor fresh frozen plasma transfusion was independently and significantly associated with patients’ overall survival (Supplementary Tables S1 and S2). This important finding indicates that the observed survival risks related to transfusion may not specifically stem from a certain blood component or transfusion administered at a specific perioperative stage. Instead, transfusion is more likely to serve as a marker reflecting critically ill patients who require any form of transfusion support—particularly multi-stage and multi-component transfusion—due to heavy tumor burden, complex surgical procedures, or poor general physical status. These findings strengthen our core viewpoint: clinical decision-making should focus not merely on “whether to transfuse” or “which blood product to transfuse”, but more importantly on the underlying conditions driving the necessity for transfusion. Furthermore, comprehensive patient blood management strategies, including optimized surgical techniques and enhanced preoperative preparation, should be implemented to reduce the demand for allogeneic blood transfusion at the source.

This study also conducted an exploratory analysis to address potential residual confounding. The results indicated that available baseline systemic inflammatory markers were not significantly associated with the need for perioperative blood transfusion (see Table S3 in the Supplementary Materials). This finding reduces the likelihood that “baseline inflammatory status is the primary confounder driving transfusion and concurrently leading to poor prognosis”, thereby indirectly supporting the main conclusion of this study: the observed survival differences are correlated with transfusion exposure itself.

This study has several limitations that should be considered when interpreting the findings. First, inherent to its retrospective, single-institutional design are selection and information biases, which may limit the generalizability of the results. The enrollment period spanned 2007 to 2015, during which evolution in surgical techniques, perioperative care, and transfusion strategies may have introduced temporal confounding, despite our matching for surgical approach. Second, key data gaps constrain causal inference. Most critically, we lack detailed data on adjuvant chemotherapy (administration, timing, dose-intensity) and post-recurrence therapy, which are established prognostic factors. Differences in these treatments between groups could theoretically account for the observed survival disparity. Furthermore, intraoperative blood loss was not recorded. Since major bleeding is the primary trigger for transfusion, our observed association inherently represents the combined effect of the physiological insult from bleeding and the therapeutic intervention of transfusion; we cannot statistically disentangle their independent contributions. Third, despite employing propensity score matching (PSM) to balance numerous baseline covariates, residual confounding remains possible. While PSM mitigated bias from many measured variables, a strong predictor for transfusion—intestinal obstruction—remained imbalanced after matching. We also could not fully account for unmeasured or poorly quantified factors such as nutritional status and the severity of specific postoperative complications (due to low event rates). However, in direct response to reviewer concerns regarding potential confounding by inflammation, supplementary analysis of available biomarkers (e.g., WBC, neutrophil counts) showed no significant association with transfusion requirement, somewhat mitigating this specific concern. Fourth, our analysis of oncological endpoints and mechanisms has inherent constraints. We focused on overall survival (OS) due to its objectivity and reliability in retrospective review, acknowledging that disease-free survival (DFS) and cancer-specific survival (CSS) could offer more direct insights into tumor biology. The accurate retrospective ascertainment of these endpoints is challenging. Moreover, we lack direct immunological biomarker data to empirically validate the proposed mechanism of transfusion-related immunomodulation (TRIM); thus, the link to TRIM remains a plausible hypothesis from the literature, not a finding of this study. Similarly, we could not analyze patterns of recurrence (e.g., local vs. distant) due to unsystematic site-specific data. Fifth, the 4-unit cutoff for transfusion volume, identified via data-driven X-tile analysis, requires validation. This threshold is derived from and applies to our single cohort; its generalizability needs confirmation in external, prospective, or multi-center studies. Despite these limitations, our findings are robust within the context of a retrospective study. The use of PSM to balance key prognostic variables, the persistence of a significant association between transfusion and worse OS in multivariate analysis, and the demonstration of a clear dose–response relationship (with stepwise risk increase from no transfusion, to low-volume [≤4 units], to high-volume [>4 units] transfusion) collectively provide strong evidence supporting perioperative blood transfusion as an independent prognostic factor. Future prospective, multi-center studies that rigorously collect detailed data on intraoperative blood loss, comprehensive transfusion parameters (all blood products), adjuvant and salvage therapies, recurrence patterns, and serial biomarkers are essential to validate these findings and elucidate the underlying biological mechanisms.

## 5. Conclusions

In conclusion, this retrospective study suggests a potential association between perioperative blood transfusion (BTF) and adverse short-term postoperative outcomes in patients with colorectal cancer (CRC), and further indicates that blood transfusion (BTF) may act as an independent prognostic factor for overall survival (OS) in this patient population, with no definitive causal inference drawn given the observational study design. Transfusion volume shows a notable correlation with clinical management and survival outcomes, highlighting its clinical relevance to perioperative decision-making. A deeper insight into these observed associations could help optimize perioperative care protocols for CRC patients, and the implementation of more conservative transfusion strategies may carry potential benefits for long-term survival. Optimizing preoperative hemoglobin levels and implementing judicious, restricted blood transfusion practices may also serve as a modifiable approach to enhance clinical outcomes, although further validation is needed. Future research is warranted to explore the underlying mechanisms linking blood transfusion (BTF) to survival and to prospectively assess the impact of targeted preoperative anemia management strategies on key clinical endpoints in CRC patients.

## Figures and Tables

**Table 1:** Pre- and Post-PSM Analysis of Clinicopathological Features in Colorectal Cancer with and without Perioperative Blood Transfusion.

Variables	Total Cohort (*n* = 1777)			Propensity Score Matched Cohort (*n* = 1048)		
	Non-BTF Group (*n* = 1048)	BTF Group (*n* = 729)	OR (Univariable)	Non-BTF Group (*n* = 524)	BTF Group (*n* = 524)	OR (Univariable)
Gender						
Female	408 (38.9%)	358 (49.1%)		237 (45.2%)	254 (48.5%)	0.88 (0.69–1.12, *p* = 0.293)
Male	640 (61.1%)	371 (50.9%)	0.66 (0.55–0.80, *p* < 0.001)	287 (54.8%)	270 (51.5%)	
Age (Mean ± SD)	60.9 ± 11.7	62.0 ± 12.9	1.01 (1.00–1.02, *p* = 0.069)	61.1 ± 12.0	62.1 ± 12.8	1.01 (1.00–1.02, *p* = 0.188)
Age group						
<60 years	405 (38.6%)	268 (36.8%)		206 (39.3%)	192 (36.6%)	
≥60 years	643 (61.4%)	461 (63.2%)	1.08 (0.89–1.32, *p* = 0.421)	318 (60.7%)	332 (63.4%)	1.12 (0.87–1.44, *p* = 0.373)
Comorbidities						
Any comorbidities	45 (4.3%)	48 (6.6%)		20 (3.8%)	36 (6.9%)	
Non-comorbidities	1003 (95.7%)	681 (93.4%)	0.64 (0.42–0.97, *p* = 0.034)	504 (96.2%)	488 (93.1%)	0.54 (0.31–0.94, *p* = 0.030)
Hb ^1^ (Mean ± SD)	134.5 ± 19.3	113.2 ± 25.5	0.96 (0.95–0.96, *p* < 0.001)	133.2 ± 19.8	115.0 ± 25.4	0.97 (0.96–0.97, *p* < 0.001)
Blood loss ^2^ (Mean ± SD)	34.4 ± 4.9	269.0 ± 274.4	1.17 (1.13–1.21, *p* < 0.001)	34.4 ± 4.9	249.7 ± 222.3	1.15 (1.10–1.20, *p* < 0.001)
Diabetes						
Diabetes	61 (5.8%)	59 (8.1%)		41 (7.8%)	40 (7.6%)	
Non-diabetes	987 (94.2%)	670 (91.9%)	0.70 (0.48–1.02, *p* = 0.061)	483 (92.2%)	484 (92.4%)	1.03 (0.65–1.62, *p* = 0.908)
Hypertension						
Hypertension	202 (19.3%)	156 (21.4%)		104 (19.8%)	115 (21.9%)	
Non-hypertension	846 (80.7%)	573 (78.6%)	0.88 (0.69–1.11, *p* = 0.272)	420 (80.2%)	409 (78.1%)	0.88 (0.65–1.19, *p* = 0.404)
Tumor.size.cm (Mean ± SD)	4.1 ± 1.8	5.4 ± 2.3	1.37 (1.30–1.45, *p* < 0.001)	4.7 ± 2.0	4.7 ± 1.9	1.02 (0.96–1.08, *p* = 0.597)
Hospitalization days (Mean ± SD)	15.5 ± 3.9	17.4 ± 4.2	1.12 (1.09–1.15, *p* < 0.001)	17.0 ± 4.2	16.8 ± 3.9	0.99 (0.96–1.02, *p* = 0.591)
T stage						
T1	39 (3.7%)	13 (1.8%)		10 (1.9%)	11 (2.1%)	
T2	149 (14.2%)	58 (8%)	1.17 (0.58–2.34, *p* = 0.663)	49 (9.4%)	54 (10.3%)	1.00 (0.39–2.56, *p* = 0.997)
T3	316 (30.2%)	219 (30%)	2.08 (1.08–3.99, *p* = 0.028)	167 (31.9%)	163 (31.1%)	0.89 (0.37–2.15, *p* = 0.791)
T4	544 (51.9%)	439 (60.2%)	2.42 (1.28–4.59, *p* = 0.007)	298 (56.9%)	296 (56.5%)	0.90 (0.38–2.16, *p* = 0.818)
N stage						
N0	692 (66%)	418 (57.3%)		316 (60.3%)	310 (59.2%)	
N1	238 (22.7%)	200 (27.4%)	1.39 (1.11–1.74, *p* = 0.004)	131 (25%)	136 (26%)	1.06 (0.79–1.41, *p* = 0.699)
N2	118 (11.3%)	111 (15.2%)	1.56 (1.17–2.07, *p* = 0.002)	77 (14.7%)	78 (14.9%)	1.03 (0.73–1.47, *p* = 0.858)
M stage						
M0	1039 (99.1%)	711 (97.5%)		515 (98.3%)	516 (98.5%)	
M1	9 (0.9%)	18 (2.5%)	2.92 (1.31–6.54, *p* = 0.009)	9 (1.7%)	8 (1.5%)	0.89 (0.34–2.32, *p* = 0.807)
pTNM stage						
I	166 (15.8%)	59 (8.1%)		52 (9.9%)	54 (10.3%)	
II	525 (50.1%)	354 (48.6%)	1.90 (1.37–2.63, *p* < 0.001)	262 (50%)	254 (48.5%)	0.93 (0.61–1.42, *p* = 0.747)
III	343 (32.7%)	295 (40.5%)	2.42 (1.73–3.38, *p* < 0.001)	200 (38.2%)	206 (39.3%)	0.99 (0.65–1.52, *p* = 0.970)
IV	14 (1.3%)	21 (2.9%)	4.22 (2.02–8.83, *p* < 0.001)	10 (1.9%)	10 (1.9%)	0.96 (0.37–2.50, *p* = 0.938)
Combined multiorgan resection						
No	1001 (95.5%)	655 (89.8%)		492 (93.9%)	489 (93.3%)	
Yes	47 (4.5%)	74 (10.2%)	2.41 (1.65–3.51, *p* < 0.001)	32 (6.1%)	35 (6.7%)	1.10 (0.67–1.81, *p* = 0.705)
Tumor site						
Left colon cancer	943 (90%)	525 (72%)		431 (82.3%)	439 (83.8%)	
Right colon cancer	105 (10%)	204 (28%)	3.49 (2.69–4.52, *p* < 0.001)	93 (17.7%)	85 (16.2%)	0.90 (0.65–1.24, *p* = 0.511)
Tumor location						
Colon cancer	310 (29.6%)	334 (45.8%)		187 (35.7%)	183 (34.9%)	
Rectal cancer	738 (70.4%)	395 (54.2%)	0.50 (0.41–0.60, *p* < 0.001)	337 (64.3%)	341 (65.1%)	1.03 (0.80–1.33, *p* = 0.796)
Surgical type						
Laparoscopic surgery	373 (35.6%)	206 (28.3%)		164 (31.3%)	174 (33.2%)	
Laparotomy	675 (64.4%)	523 (71.7%)	1.40 (1.14–1.72, *p* = 0.001)	360 (68.7%)	350 (66.8%)	0.92 (0.71–1.19, *p* = 0.509)
Pathological type						
Highly and moderately differentiated adenocarcinoma	943 (90%)	630 (86.4%)		469 (89.5%)	464 (88.5%)	
Mucinous adenocarcinoma	73 (7%)	67 (9.2%)	1.37 (0.97–1.94, *p* = 0.072)	39 (7.4%)	42 (8%)	1.09 (0.69–1.71, *p* = 0.714)
Other	8 (0.8%)	10 (1.4%)	1.87 (0.73–4.77, *p* = 0.189)	3 (0.6%)	5 (1%)	1.68 (0.40–7.09, *p* = 0.477)
Poorly differentiated adenocarcinoma	24 (2.3%)	22 (3%)	1.37 (0.76–2.47, *p* = 0.291)	13 (2.5%)	13 (2.5%)	1.01 (0.46–2.20, *p* = 0.979)
Tumor thrombus						
Non-tumor thrombus	1026 (97.9%)	708 (97.1%)		510 (97.3%)	510 (97.3%)	
Tumor thrombus	22 (2.1%)	21 (2.9%)	1.38 (0.75–2.53, *p* = 0.294)	14 (2.7%)	14 (2.7%)	1.00 (0.47–2.12, *p* = 1.000)
Family history						
No	968 (92.4%)	676 (92.7%)		--	--	
Yes	80 (7.6%)	53 (7.3%)	0.95 (0.66–1.36, *p* = 0.775)	--	--	--
Cooccurrence of other tumor						
No	1046 (99.8%)	715 (98.1%)		522 (99.6%)	521 (99.4%)	
Yes	2 (0.2%)	14 (1.9%) 1	0.24 (2.32–45.20, *p* = 0.002)	2 (0.4%)	3 (0.6%)	1.50 (0.25–9.03, *p* = 0.656)
Obstruction						
Non-obstruction	966 (92.2%)	579 (79.4%)		472 (90.1%)	433 (82.6%)	
obstruction	82 (7.8%)	150 (20.6%)	3.05 (2.29-4.07, *p*<0.001)	52 (9.9%)	91 (17.4%)	1.91 (1.32–2.75, *p*<0.0001)

^1^ Hb, preoperative hemoglobin; ^2^ Blood loss, intraoperative blood loss.

**Table 2:** Multivariate analysis of potential predictors for perioperative BTF in the total cohort and propensity score-matched cohort.

Variables	Total Cohort (*n* = 1777)			Propensity Score Matched Cohort (*n* = 1048)		
	Non-BTF Group (*n* = 1048)	BTF Group (*n* = 729)	OR (Multivariable)	Non-BTF Group (*n* = 524)	BTF Group (*n* = 524)	OR (Multivariate)
Gender						
Female	408 (38.9%)	358 (49.1%)		237 (45.2%)	254 (48.5%)	
Male	640 (61.1%)	371 (50.9%)	0.56 (0.45–0.70, *p* < 0.001)	287 (54.8%)	270 (51.5%)	0.85 (0.66–1.09, *p* = 0.209)
Age(Mean ± SD)	60.9 ± 11.7	62.0 ± 12.9	1.01 (0.99–1.02, *p* = 0.363)	60.9 ±11.9	62.0 ±12.8	1.01 (0.99–1.03, *p* = 0.195)
Age group						
<60 years	405 (38.6%)	268 (36.8%)		206 (39.3%)	193 (36.8%)	
≥60 years	643 (61.4%)	461 (63.2%)	0.98 (0.68–1.42, *p* = 0.928)	318 (60.7%)	331 (63.2%)	0.88 (0.57–1.35, *p* = 0.562)
Hb ^1^ (Mean ± SD)	134.5 ± 19.3	113.2 ± 25.5	0.96 (0.95–0.97, *p* < 0.001)	133.2 ± 19.8	115.0 ± 25.4	0.97 (0.96–0.98, *p* < 0.001)
Blood loss ^2^ (Mean ± SD)	34.4 ± 4.9	269.0 ± 274.4	1.12 (1.08–1.16, *p* < 0.001)	34.4 ± 4.9	249.7 ± 222.3	1.12 (1.08–1.17, *p* < 0.001)
Comorbidities						
Any comorbidities	45 (4.3%)	48 (6.6%)		19 (3.6%)	36 (6.9%)	
Non-comorbidities	1003 (95.7%)	681 (93.4%)	0.61 (0.38–0.99, *p* = 0.045)	505 (96.4%)	488 (93.1%)	0.51 (0.28–0.94, *p* = 0.030)
Diabetes						
Diabetes	61 (5.8%)	59 (8.1%)		39 (7.4%)	40 (7.6%)	
Non-diabetes	987 (94.2%)	670 (91.9%)	1.04 (0.68–1.60, *p* = 0.856)	485 (92.6%)	484 (92.4%)	1.10 (0.68–1.77, *p* = 0.710)
Hypertension						
Hypertension	202 (19.3%)	156 (21.4%)		105 (20%)	115 (21.9%)	
Non-hypertension	846 (80.7%)	573 (78.6%)	0.93 (0.71–1.22, *p* = 0.589)	419 (80%)	409 (78.1%)	0.96 (0.70–1.32, *p* = 0.822)
Tumor size(cm) (Mean ± SD)	4.1 ± 1.8	5.4 ± 2.3	1.29 (1.21–1.38, *p* < 0.001)	4.7 ±2.0	4.7 ±1.9	1.02 (0.95–1.10, *p* = 0.512)
Hospitalization days (Mean ± SD)	15.5 ± 3.9	17.4 ± 4.2	1.12 (1.09–1.15, *p* < 0.001)	17.0 ±4.2	16.8 ±3.9	0.98 (0.95–1.02, *p* = 0.322)
T stage						
T1	39 (3.7%)	13 (1.8%)		10 (1.9%)	11 (2.1%)	
T2	149 (14.2%)	58 (8%)	0.84 (0.40–1.78, *p* = 0.646)	49 (9.4%)	54 (10.3%)	0.94 (0.36–2.45, *p* = 0.899)
T3	316 (30.2%)	219 (30%)	0.78 (0.29–2.10, *p* = 0.630)	167 (31.9%)	163 (31.1%)	0.55 (0.15–1.99, *p* = 0.363)
T4	544 (51.9%)	439 (60.2%)	0.61 (0.23–1.63, *p* = 0.327)	298 (56.9%)	296 (56.5%)	0.52 (0.14–1.86, *p* = 0.312)
N stage						
N0	692 (66%)	418 (57.3%)		316 (60.3%)	310 (59.2%)	
N1	238 (22.7%)	200 (27.4%)	0.88 (0.23–3.37, *p* = 0.858)	131 (25%)	136 (26%)	0.95 (0.18–4.89, *p* = 0.950)
N2	118 (11.3%)	111 (15.2%)	1.01 (0.27–3.79, *p* = 0.988)	77 (14.7%)	78 (14.9%)	0.91 (0.18–4.61, *p* = 0.909)
M stage						
M0	1039 (99.1%)	711 (97.5%)		515 (98.3%)	516 (98.5%)	
M1	9 (0.9%)	18 (2.5%)	1.38 (0.32–5.93, *p* = 0.666)	9 (1.7%)	8 (1.5%)	0.68 (0.12–3.76, *p* = 0.662)
pTNM stage						
I	166 (15.8%)	59 (8.1%)		52 (9.9%)	54 (10.3%)	
II	525 (50.1%)	354 (48.6%)	1.43 (0.63–3.23, *p* = 0.390)	262 (50%)	254 (48.5%)	1.53 (0.55–4.25, *p* = 0.417)
III	343 (32.7%)	295 (40.5%)	2.30 (0.52–10.13, *p* = 0.271)	200 (38.2%)	206 (39.3%)	1.62 (0.26–10.20, *p* = 0.610)
IV	14 (1.3%)	21 (2.9%)	1.80 (0.33–9.89, *p* = 0.500)	10 (1.9%)	10 (1.9%)	1.81 (0.21–15.65, *p* = 0.588)
Combined multiorgan resection						
No	1001 (95.5%)	655 (89.8%)		492 (93.9%)	489 (93.3%)	
Yes	47 (4.5%)	74 (10.2%)	1.73 (1.05–2.86, *p* = 0.032)	32 (6.1%)	35 (6.7%)	1.24 (0.68–2.24, *p* = 0.481)
Tumor site						
Left colon cancer	943 (90%)	525 (72%)		431 (82.3%)	439 (83.8%)	
Right colon cancer	105 (10%)	204 (28%)	2.57 (1.80–3.68, *p* < 0.001)	93 (17.7%)	85 (16.2%)	0.82 (0.54–1.26, *p* = 0.369)
Tumor location						
Colon cancer	310 (29.6%)	334 (45.8%)		187 (35.7%)	183 (34.9%)	
Rectal cancer	738 (70.4%)	395 (54.2%)	0.87 (0.65–1.18, *p* = 0.375)	337 (64.3%)	341 (65.1%)	0.98 (0.70–1.39, *p* = 0.932)
Surgical type						
Laparoscopic surgery	373 (35.6%)	206 (28.3%)		164 (31.3%)	174 (33.2%)	
Laparotomy	675 (64.4%)	523 (71.7%)	0.98 (0.77–1.23, *p* = 0.845)	360 (68.7%)	350 (66.8%)	0.92 (0.70–1.21, *p* = 0.565)
Pathological type						
Highly and moderately differentiated adenocarcinoma	943 (90%)	630 (86.4%)		471 (89.9%)	464 (88.5%)	
Mucinous adenocarcinoma	73 (7%)	67 (9.2%)	0.96 (0.64–1.44, *p* = 0.849)	37 (7.1%)	42 (8%)	1.16 (0.72–1.89, *p* = 0.544)
Other	8 (0.8%)	10 (1.4%)	1.15 (0.37–3.60, *p* = 0.815)	3 (0.6%)	5 (1%)	1.70 (0.39–7.39, *p* = 0.479)
Poorly differentiated adenocarcinoma	24 (2.3%)	22 (3%)	0.75 (0.38–1.49, *p* = 0.415)	13 (2.5%)	13 (2.5%)	0.92 (0.40–2.11, *p* = 0.848)
Tumor thrombus						
Non-tumor thrombus	1026 (97.9%)	708 (97.1%)		510 (97.3%)	510 (97.3%)	
Tumor thrombus	22 (2.1%)	21 (2.9%)	0.97 (0.49–1.91, *p* = 0.923)	14 (2.7%)	14 (2.7%)	1.03 (0.47–2.25, *p* = 0.949)
Family history						
No	968 (92.4%)	676 (92.7%)		NA	NA	
Yes	80 (7.6%)	53 (7.3%)	1.05 (0.70–1.58, *p* = 0.810)	NA	NA	
Cooccurrence of other tumor						
No	1046 (99.8%)	715 (98.1%)		522 (99.6%)	521 (99.4%)	
Yes	2 (0.2%)	14 (1.9%) 5	0.87 (1.15–30.01, *p* = 0.033)	2 (0.4%)	3 (0.6%)	1.11 (0.18–6.96, *p* = 0.909)
Obstruction						
Non-obstruction	966 (92.2%)	579 (79.4%)		472 (90.1%)	433 (82.6%)	
Obstruction	82 (7.8%)	150 (20.6%)	2.06 (1.48–2.86, *p* < 0.001)	52 (9.9%)	91 (17.4%)	2.06 (1.40–3.02, *p* < 0.001)

^1^ Hb, preoperative hemoglobin; ^2^ Blood loss, intraoperative blood loss.

**Table 3:** Univariate analysis of prognostic factors for overall survival following radical resection of colorectal cancer in the total cohort and propensity score-matched cohort.

Variables	Total Cohort		Propensity Score Matched Cohort	
	*p* Value	HR (95% CI)	*p* Value	HR (95% CI)
BTF	<0.001	1.816 (1.473–2.239)	0.001	1.563 (1.193–2.048)
Gender	0.316	0.899 (0.729–1.108)	0.467	0.906 (0.694–1.182)
Age	0.593	1.002 (0.994–1.011)	0.891	0.999 (0.989–1.010)
Age group	0.635	0.949 (0.765–1.178)	0.726	0.952 (0.721–1.256)
Comorbidities	0.976	1.007 (0.627–1.619)	0.765	0.915 (0.511–1.638)
Diabetes	0.230	0.783 (0.525–1.167)	0.431	0.819 (0.499–1.345)
Hypertension	0.796	0.966 (0.742–1.257)	0.667	0.929 (0.665–1.299)
Tumor size (cm)	<0.001	1.127 (1.082–1.174)	0.007	1.085 (1.023–1.151)
Hospitalization days	0.107	1.020 (0.996–1.045)	0.871	0.997 (0.965–1.030)
Combined multiorgan resection	0.022	1.511 (1.061–2.152)	0.699	1.106 (0.665–1.840)
Tumor site	0.039	1.320 (1.014–1.718)	0.963	1.009 (0.702–1.448)
Tumor location	0.008	0.748 (0.604–0.926)	0.791	1.039 (0.782–1.380)
Surgical type	0.022	1.320 (1.041–1.675)	0.305	1.168 (0.868–1.570)
Tumor thrombus	0.042	1.775 (1.020–3.090)	0.014	2.139 (1.166–3.924)
Family history	0.086	0.653 (0.401–1.063)	--	--
Cooccurrence of other tumor	0.241	0.309 (0.043–2.199)	0.970	0.963 (0.135–6.872)
Pathological type				
Mucinous adenocarcinoma	<0.001	2.035 (1.494–2.773)	<0.001	2.150 (1.450–3.188)
Other	0.018	2.478 (1.170–5.246)	<0.001	7.024 (2.877–17.150)
Poorly differentiated adenocarcinoma	<0.001	3.578 (2.295–5.577)	<0.001	4.056 (2.305–7.137)
T stage				
T2	0.861	1.118 (0.3213–3.890)	0.570	1.821 (0.231–14.380)
T3	0.055	3.084 (0.9769–9.737)	0.184	3.816 (0.529–27.540)
T4	0.007	4.825 (1.545–15.068)	0.082	5.734 (0.802–40.980)
N stage				
N1	<0.001	2.788 (2.148–3.617)	<0.001	2.494 (1.762–3.528)
N2	<0.001	6.853 (5.323–8.821)	<0.001	7.259 (5.280–9.979)
M stage	<0.001	3.186 (1.746–5.815)	0.031	2.447 (1.086–5.513)
Obstruction	<0.001	2.675 (2.107–3.396)	<0.001	2.791 (2.071–3.759)
pTNM stage				
II	0.017	1.980 (1.133–3.460)	0.175	1.718 (0.786–3.759)
III	<0.001	7.086 (4.128–12.160)	<0.001	6.348 (2.972–13.561)
IV	<0.001	9.564 (4.490–20.370)	<0.001	9.746 (3.529–26.916)

**Table 4:** Univariate analysis of prognostic factors for overall survival following radical resection of colorectal cancer.

Variables	*p* Value	HR (95% CI)
BTF group		
SBTF	<0.001	1.642 (1.296–2.080)
MBTF	<0.001	2.222 (1.675–2.947)
Obstruction	<0.001	2.675 (2.107-3.396)
Gender	0.316	0.899 (0.729–1.108)
Age	0.593	1.002 (0.994–1.011)
Age group	0.635	0.949 (0.765–1.178)
Comorbidities	0.976	1.007 (0.627–1.619)
Diabetes	0.230	0.783 (0.525–1.167)
Hypertension	0.796	0.966 (0.742–1.257)
Tumor size cm	<0.001	1.127 (1.082–1.174)
Hospitalization days	0.107	1.020 (0.996–1.045)
Combined multiorgan resection	0.022	1.511 (1.061–2.152)
Tumor site	0.039	1.320 (1.014–1.718)
Tumor location	0.008	0.748 (0.604–0.926)
Surgical type	0.022	1.320 (1.041–1.675)
Tumor thrombus	0.042	1.775 (1.020–3.090)
Family history	0.086	0.653 (0.401–1.063)
Cooccurrence of other tumor	0.241	0.309 (0.043–2.199)
Pathological type		
Mucinous adenocarcinoma	<0.001	2.035 (1.494–2.773)
Other	0.018	2.478 (1.170–5.246)
Poorly differentiated adenocarcinoma	<0.001	3.578 (2.295–5.577)
T stage		
T2	0.861	1.118 (0.321–3.890)
T3	0.055	3.084 (0.977–9.737)
T4	0.007	4.825 (1.545–15.068)
N stage		
N1	<0.001	2.788 (2.148–3.617)
N2	<0.001	6.853 (5.323–8.821)
M stage	0.000	3.186 (1.746–5.815)
pTNM stage		
II	0.017	1.980 (1.133- 3.460)
III	<0.001	7.086 (4.128–12.160)
IV	<0.001	9.564 (4.490–20.370)

## Data Availability

Data is unavailable due to privacy restrictions.

## Supplementary Materials

The following supporting information can be downloaded at: https://www.mdpi.com/article/10.3390/cancers18081198/s1, Table S1: Logistic Regression Analysis of Postoperative Hematological Parameters with Blood Transfusion (BTF) Requirement; Table S2: Association Between Perioperative Transfusion Timing of Red Blood Cells and Overall Survival; Table S3: Association Between Perioperative Transfusion Timing of Fresh Frozen Plasma and Overall Survival.

## Abbreviations

The following abbreviations are used in this manuscript:

CRC	Colorectal cancer
BTF	Blood transfusion
TRIM	Transfusion-related immune modulation
PSM	Propensity score matching
CI	Confidence interval
OS	Overall survival
HR	Hazard ratio
SD	Standard deviation
SBTF	Small-volume blood transfusion
MBTF	Massive-volume blood transfusion
OR	Odds ratio
CVP	Central venous pressure
DFS	Disease-free survival
RFS	Recurrence-free survival
