# Supplementary material for: Perioperative Blood Transfusion Impairs Overall Survival Following Radical Resection for Colorectal Cancer: A Propensity Score-Matched Analysis
Source: Cancers (Basel). 2026 Apr 9;18(8):1198. doi: 10.3390/cancers18081198 (PMC13115186; doi:10.3390/cancers18081198)
Supplement: Supplementary file 1 [file cancers-18-01198-s001.zip › cancers-4204350-supplementary.pdf]

Table S1. Logistic Regression Analysis of Postoperative Hematological Parameters with Blood Transfusion(BTF) Requirement.

Variable	β (SE)	OR (95% CI)	z Value	p Value
WBC count				
Neutropenia	0.212 (0.257)	1.236 (0.746–2.045)	0.823	0.410
Normal range	0.022 (0.251)	1.023 (0.625–1.674)	0.089	0.929
Neutrophil count				
Neutrophilia	0.191 (0.108)	1.210 (0.980–1.494)	1.773	0.076
Leukocytosis	0.207 (0.112)	1.229 (0.988–1.530)	1.850	0.064
Normoleukocytosis	0.097 (0.160)	1.102 (0.805–1.507)	0.606	0.545
NWR	0.050 (0.308)	1.052 (0.576–1.920)	0.164	0.870

Abbreviation: NWR, neutrophil-to-white blood cell ratio.

Table S2. Association Between Perioperative Transfusion Timing of Red Blood Cells and Overall Survival.

Variables	p.value	HR (95% CI)
Transfusion Periods		
Intraoperative	0.563	1.204 (0.641–2.262)
Postoperative	0.549	0.888 (0.602–1.310)
Preoperative	0.576	0.673 (0.167–2.703)

Table S3. Association Between Perioperative Transfusion Timing of Fresh Frozen Plasma and Overall Survival.

Variables	p.value	HR (95% CI)
Transfusion Periods of Plasma		
Intraoperative	0.314	0.660 (0.294–1.482)
Postoperative	0.483	0.872 (0.595–1.278)
Preoperative	0.444	0.464 (0.065–3.309)
